# Flow Cytometry-Based Quantification of Neutrophil Extracellular Traps Shows an Association with Hypercoagulation in Septic Shock and Hypocoagulation in Postsurgical Systemic Inflammation—A Proof-of-Concept Study

**DOI:** 10.3390/jcm9010174

**Published:** 2020-01-08

**Authors:** Emmanuel Schneck, Franziska Mallek, Julia Schiederich, Emil Kramer, Melanie Markmann, Matthias Hecker, Natascha Sommer, Norbert Weissmann, Oleg Pak, Gabriela Michel, Andreas Hecker, Winfried Padberg, Andreas Boening, Michael Sander, Christian Koch

**Affiliations:** 1 Department of Anesthesiology, Operative Intensive Care Medicine and Pain Therapy, Justus Liebig University of Giessen, Rudolf-Buchheim-Strasse 7, 35392 Giessen, Germany; Franziska.Mallek@med.uni-giessen.de (F.M.); julia.schiederich@web.de (J.S.); Emil.V.Kramer@med.uni-giessen.de (E.K.); Melanie.Markmann@chiru.med.uni-giessen.de (M.M.); michael.sander@chiru.med.uni-giessen.de (M.S.); christian.koch@chiru.med.uni-giessen.de (C.K.); 2 German Centre for Infection Research (DZIF), Partner Site Giessen-Marburg-Langen, 35392 Giessen, Germany; 3 Department of Pulmonary Medicine, University of Giessen and Marburg Lung Center (UGMLC), University Hospital of Giessen, Justus-Liebig-University of Giessen, 35392 Giessen, Germany; Matthias.Hecker@innere.med.uni-giessen.de (M.H.); Natascha.Sommer@innere.med.uni-giessen.de (N.S.); Norbert.Weissmann@innere.med.uni-giessen.de (N.W.); Oleg.Pak@innere.med.uni-giessen.de (O.P.); 4 Department of Immunology and Transfusion Medicine, Langhans Street 7, 35392 Giessen, Germany; Gabriela.Michel@immunologie.med.uni-giessen.de; 5 Department of General and Thoracic Surgery, University Hospital of Giessen, Justus-Liebig-University of Giessen, 35392 Giessen, Germany; andreas.hecker@chiru.med.uni-giessen.de (A.H.); Winfried.Padberg@chiru.med.uni-giessen.de (W.P.); 6 Department of Adult and Pediatric Cardiovascular Surgery, Giessen University Hospital, 35392 Giessen, Germany; Andreas.Boening@chiru.med.uni-giessen.de

**Keywords:** inflammation, sepsis, coagulopathy, cardiopulmonary bypass, major abdominal surgery

## Abstract

This proof-of-concept study aimed to evaluate a novel method of flow cytometry-based quantification of neutrophil extracellular traps (NETs) in septic shock patients and to identify possible interactions between the number of free-circulating NETs and alterations of the coagulatory system. Patients suffering from septic shock, a matched control group (CTRL), and patients suffering from systemic inflammation after cardiac (CABG) or major abdominal surgery (MAS) were enrolled in this prospective proof-of-concept study. Compared to the matched controls, free-circulating NETs were significantly elevated in septic shock and postsurgical patients (data are presented in median (IQR)); septic shock: (2.7 (1.9–3.9); CABG: 2.7 (2.1–3.7); MAS: 2.7 (2.1–3.9); CTRL: 1.6 (1–2); CTRL vs. septic shock: *p* = 0.001; CTRL vs. CABG: *p* < 0.001; CTRL vs. MAS: *p* < 0.001). NETs correlated positively with FIBTEM mean clot firmness (MCF) in septic shock patients (*r* = 0.37, *p* < 0.01) while they correlated negatively in surgical patients (CABG: *r* = −0.28, *p* < 0.01; MAS: *r* = −0.25, *p* = 0.03). Flow-cytometric quantification of NETs showed a significant increase in free-circulating NETs under inflammatory conditions. Furthermore, this study hints to an association of the number of NETs with hypercoagulation in septic shock patients and hypocoagulation in surgery-induced inflammation.

## 1. Introduction

Despite tremendous efforts to develop new diagnostic and therapeutic approaches, septic shock still remains associated with high mortality. Particularly, patients suffering from septic coagulopathy are considered to be at high risk for limited outcome [1,2]. This is recognized by the Sepsis-3 definition, which highlights that the host´s pathophysiological reactions to a pathogen are determined by the severity of organ failure [3]. Furthermore, tissue hypoperfusion is defined as the main symptom in septic shock; it is caused by vasoplegia, endothelial damage, and leakage, as well as capillary thrombotic occlusion [2]. Next to sepsis-induced alterations of the renal, hepatic, cardiovascular, respiratory, and central venous system, coagulopathies display a common and hazardous complication of sepsis [4,5]. Lyons et al. investigated 6148 septic patients and identified the presence of sepsis-associated coagulopathy (SAC) as an independent predictor for increased mortality [4]. Until today, despite ambitious approaches, no specific treatment of SAC could be successfully established [6,7,8]. For this reason, early detection of sepsis and SAC are crucial for the survival of patients. Diagnostic management should be based on the combined scoring of clinical signs of coagulopathy and blood coagulation parameters [1]. However, despite innovative approaches, there is still no specific biomarker for the early detection of SAC available for clinical routine use [9,10,11,12,13]. By investigating the underlying interactions between the innate immune and coagulatory systems, immunothrombosis has been identified as an important trigger of systemic inflammation and offers new diagnostic and therapeutic approaches for the management of septic coagulopathy [14,15,16,17].

Neutrophil extracellular traps (NETs) were identified as major players in immunothrombosis [16,17]. Consisting of nucleic acids, histones, and granule contents, such as myeloperoxidase (MPO) and neutrophil elastase, NETs are released by neutrophils. Once set free in the capillary vasculature, they form web-like formations that trap pathogens and closely interact with platelets. The capture of pathogens decelerates their spread and concentrates neutrophil antimicrobial activity. However, NETs might contribute to negative effects, such as excessive activation of coagulation, which eventually results in disseminated intravascular coagulation [18,19,20]. Several determinants lead to an NET-induced activation of the coagulatory system: Due to their polyanionic surface, NETs activate the intrinsic plasmatic system while the extrinsic pathway is stimulated by tissue factor presentation of NETs and platelets are strongly activated by DNA/histones complexes [21,22,23,24]. However, despite an increasing number of studies hinting towards a procoagulatory effect of NETs, the clinical relevance of these findings remains controversial [18,19,23,25].

Elevated plasma levels of NETs were first identified in autoimmune and cancerous diseases, but current studies proved their crucial role in the development of sepsis [26,27,28,29,30,31,32]. Furthermore, some studies indicate that NETs are also elevated in the peripheral plasma of patients suffering from systemic inflammation after major surgery [27,33,34,35]. Nevertheless, even though the amount of free-circulating NETs is increased in sepsis and surgery-induced systemic inflammation, it remains unknown if NETs offer discriminative value in distinguishing between septic shock and postsurgical systemic inflammation [31,36]. Particularly, in the early postsurgical phase, discrimination of severe infectious complications from sterile postsurgical inflammation remains challenging in the clinical routine [37,38].

Since NETs may offer an opportunity for novel diagnostic and therapeutic approaches, the need for robust and clinically feasible quantification of NETs arises. Until today, fluorescence microscopy remains the most established method for NET quantification; however, this method has some limitations. First, it only analyzes an abstract of a sample; second, it is not feasibly for daily intensive care unit (ICU) routine; and third, it is highly dependent on expert scientists’ skills and interpretations. In contrast, flow cytometry offers fast and reliable counting of a high number of neutrophils. In 2015, two methods of flow cytometry-based NET quantification were published by two separate research facilities. While Zhao et al. established a combination of high-speed multi-spectral imaging and morphometric image analysis, Gavillet et al. used a direct flow cytometry-based assay for NET identification and quantification [39,40]. Although both approaches are able to count a high number of cells and perform morphological analysis, they also feature some limitations. First, they are highly specialized and require expert knowledge, which makes them less suitable for research in the clinical routine. Second, the appropriate gating strategy to identify NET-releasing neutrophils is still under discussion [41,42]. In 2018, Lee et al. published an optimized method of Gavillet et al.’s protocol, which aimed to quantify NET-releasing neutrophils by using whole blood probes without cell fixation [43]. Therefore, this method seems more feasible for implementation in an ICU.

This proof-of-concept study aimed to investigate the flow cytometry-based quantification of NETs in a clinical intensive care setting. First, we hypothesized that flow cytometry-based quantification of NETs is able to distinguish between matched control and septic shock patients as well as patients suffering from surgery-induced inflammation. Second, we aimed to identify possible interactions between the number of free-circulating NETs and coagulatory dysfunctions using thrombelastography as a practicable solution for use in the clinical routine.

## 2. Experimental Section

### 2.1. Study Design

This single-center, prospective, observational proof-of-concept study included 80 patients who were enrolled from October 2018 to March 2019 at the University Hospital of Giessen. The study was approved by the local ethics committee (Justus-Liebig-University of Giessen, trial code: 86/18) and registered in the German Clinical Trials Register (trial code: DRKS00013584). The methods and results are presented in accordance with the Strengthening the Reporting of Observational Studies in Epidemiology (STROBE) guidelines and the Declaration of Helsinki.

Patients of legal age were enrolled at the University Hospital of Giessen after signing an informed consent form. If patients were not able to consent to the study, consent was obtained through their legal representatives. General exclusion criteria comprised an age <18 years, current pregnancy or nursing, history of recent severe trauma, autoimmune disease, severe valvular heart disease, active hematological disease, the need for immunomodulatory medication, or having undergone therapy with extracorporeal life support or renal replacement therapy prior to study inclusion. Septic shock patients must have met the Sepsis-3 definition of septic shock [3]. Cardiac surgical patients underwent coronary artery bypass graft surgery (CABG) while visceral surgical patients received major abdominal surgery (MAS), such as the Whipple procedure, oncological esophageal and gastric resection, or colectomy. Surgical patients had to meet at least two criteria of the systemic inflammatory response syndrome (SIRS) within 24 h after surgery [44]. Since no baseline values were achievable in septic patients, control (CTRL) patients were matched to them. CTRL patients were matched to septic patients based on age and gender as well as pre-existing conditions, such as a history of malignant solid or hematologic diseases, arteriosclerosis (coronary, cerebral, or peripheral artery disease), chronic renal disease, or diabetes mellitus.

Blood was collected shortly prior to surgery as well as immediately, 24, and 72 h postoperatively in surgical patients. From septic shock patients, blood was first drawn after admission to the ICU and again after diagnosis of septic shock, followed by blood collections after 24 and 72 h. CTRL patients were asked for blood donation at a single time point. Blood was collected in ethylenediaminetetraacetic acid (EDTA) for flow cytometry, fluorescence microscopy, and ELISA, while citrate tubes were used for thrombelastometry and hirudin tubes for whole blood ristocetin-induced platelet impedance aggregometry. Plasma samples were stored at −80 °C and only thawed once for the ELISA analyses. Clinical data were extracted from the local patient data management system (IMESO GmbH, Giessen, Germany).

### 2.2. Flow Cytometry

The flow cytometry protocol was adapted from the protocol published by Lee et al. [43]. In brief, 100 µL of whole blood were incubated with 10 µg immunoglobin G (IgG, 10% solution, Grifols, Barcelona, Spain) for 10 min in order to eliminate unspecific binding sites. Afterwards, 5 µL of directly-conjugated anti-H3-Histone antibody (Alexa Fluor 647 Anti-Histone, BioLegend, San Diego, CA, USA) were incubated for 30 min in darkness at room temperature followed by the application of 5 µL of anti-human cluster of differentiation (CD) 15^+^- (Pacific Blue^TM^ anti-human CD15 antibody, BioLegend, San Diego, CA, USA) and 10 µL of myeloperoxidase-(MPO)-antibody (ab11729, Abcam, Cambridge, UK). The mixture was then incubated again for 30 min at room temperature in darkness. In the next step, 1 mL of lysis buffer (1:10 dilution, BD Pharm Lyse^TM^, Frankin Lakes, NJ, USA) was incubated for 10 min under the same conditions for red blood cell lysis. Next, 1 mL of 2% bovine serum albumin in phosphate-buffered saline (PBS) was applicated followed by centrifugation (200 g for 10 min). Following this, the supernatant was separated and 300 µL of PBS were applied. Impairment of membrane integrity by red blood cell lysis was checked in healthy controls using the application of 7-aminoactinomycin (7-ADD, application five minutes prior to measurement, #559925, BD Biosciences, Franklin Lakes, NJ, USA, Supplement 1 in Supplementary Materials). Furthermore, in preliminary tests, a positive control was performed by stimulating the whole blood of healthy subjects with phorbol 12-myristate 13-acetate (100 nmol/L, PMA, Sigma, St. Louis, MO, USA) for 4 h (Supplement 2 in Supplementary Materials) [43,45].

**Figure 1:**
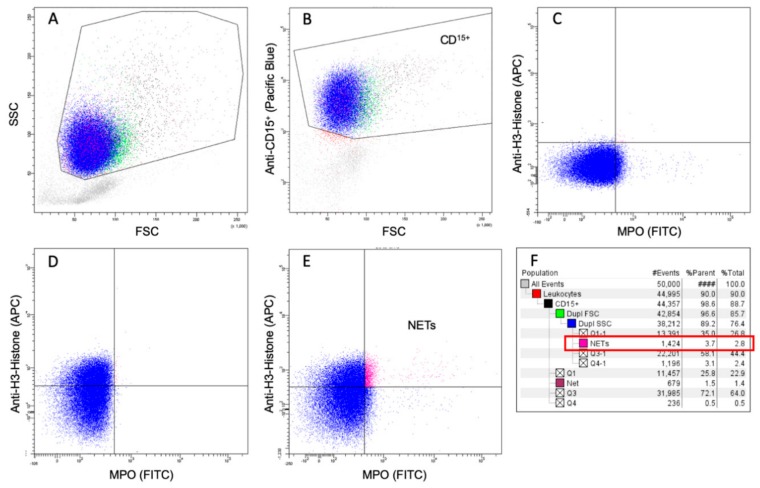
Description of the flow-cytometric gating strategy. First, leucocytes were targeted (**A**) and neutrophils identified as CD15^+^-cells (**B**). Second, the fluorescence-minus-one (FMO) technique was used to set the gating borders of MPO- and anti-H3-Histone-antibody (**C**,**D**) and last, MPO- and anti-H3-Histone-positive cells were defined as surrogates for NETs (**E**). Results are shown as the percentage of gated neutrophils (**F**, red box). Abbreviations: APC: Allophycocyanin; CD: Cluster of Differentiation; FITC: Fluorescein isothiocyanate; FSC: Forward Scatter; MPO: Myeloperoxidase; NETs: Neutrophil Extracellular Traps; SSC: Side Scatter.

BD FACS Canto II and BD FACSDIVA software (version 6.1.3, Franklin Lakes, NJ, USA) were used for flow cytometric analysis. In order to avoid detection bias, samples were blinded for flow cytometry. The gating strategy involved three steps: First, leucocytes were targeted, and neutrophils were identified as CD15^+^-cells. Second, the fluorescence-minus-one (FMO) technique was used to set the gating borders of MPO- and anti-H3-Histone-antibody. Finally, MPO- and anti-H3-Histone-antibody-positive cells were defined as surrogates for NETs (Figure 1). In order to exclude neutrophil aggregates, we checked for neutrophil duplicates. Furthermore, we quantified NETs with isotype controls in preliminary experiments to exclude relevant background fluorescence signals and found comparable results to the original description of Lee et al. (Supplement 2 in Supplementary Materials) [43]. Results were shown as the percentage of NETs for all gated neutrophils. Gating results ≤0.5% were excluded due to the impossibility of exact discrimination.

### 2.3. Fluorescence Microscopy

In order to validate MPO- and anti-H3-Histone-antibody-positive cells as NETs surrogates, confocal fluorescence microscopy was performed. In total, 100 µL of whole blood were incubated with 10 µL of PMA (100 nmol/L) for four hours, followed by red blood cell lysis (incubation of 1 mL of Pharm Lyse^TM^ (1:10 dilution) for 10 min). Afterwards, lysis was stopped using 1 mL of PBS, and centrifugation was performed with 200 g at 20 °C for 10 min. Then, the supernatant was decanted and 3 min of Cytospin^®^ (Cellspin 1, Tharmac, Wiesbaden, Germany) centrifugation was used for fixation on cover slips followed by staining with 5 µL of anti-H3-histone (Alexa Fluor 647 Anti-Histone, BioLegend, San Diego, CA, USA), 5 µL of anti-human-CD15 (Pacific Blue^TM^ anti-human CD15 antibody, BioLegend, San Diego, CA, USA), and 10 µL of MPO-antibody (ab11729, Abcam, Cambridge, UK) was performed. After incubation for 30 min in darkness, samples were washed with PBS and analyzed with fluorescence microscopy (Leica TCS SP5, Wetzlar, Germany) (Supplement 3 in Supplementary Materials).

### 2.4. ELISA

ELISA analyses included measurements of High-Mobility-Group-Protein B1 (HMGB1, Human HMGB1 ELISA Kit, Aviva Systems Biology, San Diego, CA, USA), MPO (Human MPO Instant ELISA, eBioscience, Frankfurt a.M., Germany), and interleukin-8 (IL-8, Human IL-8/CXCL8 Quantikine HS ELISA, R&D Systems, Minneapolis, MN, USA); these analyses were performed according to the manufacturer’s instructions. The optical density of samples was measured with the recommended absorbance (HMGB1: 450 nm; MPO: 450 nm, IL-8: 490 nm) and analyzed using an automated plate reader (Epoch; BioTek Instruments GmbH, Heilbronn, Germany).

### 2.5. Coagulatory Analyses

Point-of-care devices were used for thrombelastography (ROTEM^®^, Matel Medizintechnik, Hausmannstaetten, Austria) and whole blood ristocetin-induced platelet impedance aggregometry (Multiplate^®^, Roche Diagnostics, Rotkreuz, Switzerland), while the results of all other coagulatory tests were derived from the local clinical laboratory routine. Thrombelastographic assays included NATEM^®^, INTEM^®^, FIBTEM^®^, and EXTEM^®^, while the clotting and clot formation time (CT and CFT, respectively, both in seconds), mean clot firmness (MCF, mm), and lysis index after 60 min (LI60, %) were analyzed. Whole blood ristocetin-induced platelet impedance aggregometry was performed after stimulation by adenosindiphosphate (ADPtest^®^), thrombin-receptor activator protein 6 (TRAPtest^®^), and arachidonic acid (ASPItest^®^). Furthermore, the severity of coagulopathy was scored using the SAC score [46].

### 2.6. Statistical Analysis

First, values were tested for normal distribution using the Shapiro–Wilk test. Parametric data were described with mean and standard deviation while the median and interquartile range were used for non-parametric data. For statistical analysis of differences in the amount of free-circulating NETs between the study groups, the Kruskal–Wallis test was performed followed by the Wilcoxon test for analysis of intergroup differences. The analysis of the variation in the number of free-circulating NETs according to the timepoint was accomplished by applying the Friedman test followed by the pairwise Wilcox test for an analysis of the differences between timepoints within each study group. For this purpose, septic shock patients were compared to their matched controls. The correlation of NETs with respective parameters was expressed as a Pearson´s correlation coefficient. Clinical data, laboratory routine data, and experimental data were recorded in an external database (Microsoft Excel, Redmond, WA, USA). Data were processed and analyzed using R statistical software version 3.4.2 (www.r-project.org).

## 3. Results

Patient characteristics are shown in Table 1. All data are presented as median (interquartile range (IQR)).

### 3.1. Quantification of Free Circulating NETs

**Figure 2:**
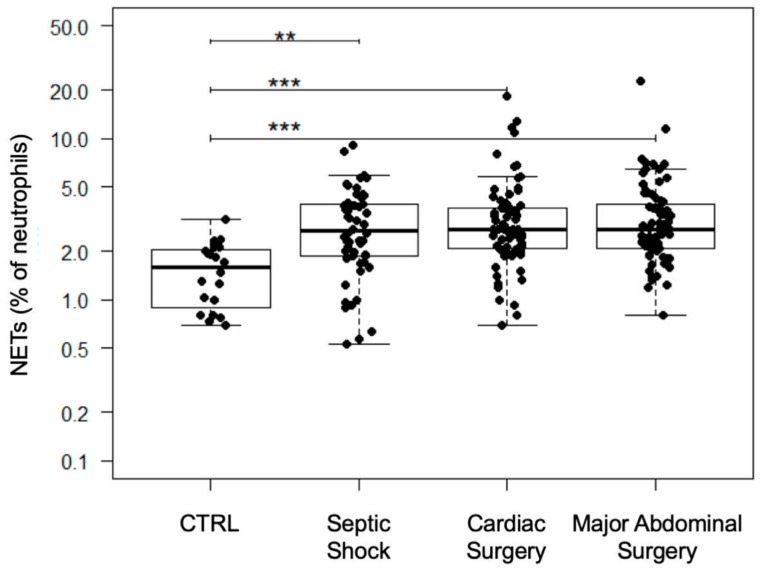
Results of the NET quantification of the study groups. With the exception of preoperative values, all time points per group were summarized. Results are shown in boxplot diagrams. Asterisks display the degree of statistical significance: **: *p* < 0.01; ***: *p* < 0.001. Abbreviations: NETs: Neutrophil Extracellular Traps.

**Figure 3:**
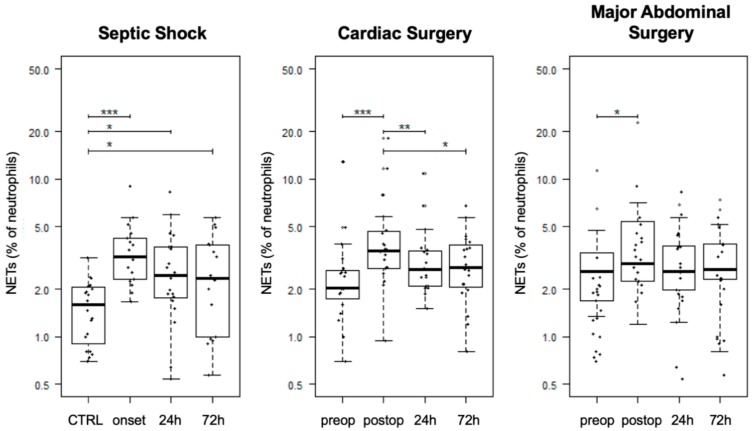
Time courses of free-circulating NETs. Results are shown in boxplot diagrams. Asterisks display the degree of statistical significance: *: *p* < 0.05; **: *p* < 0.01; ***: *p* < 0.001. Abbreviations: CTRL: Control group; NETs: Neutrophil Extracellular Traps.

Compared to matched control patients, levels of free-circulating NETs were statistically significantly elevated in all patient samples independently of the study group and time point (Figure 2, Table 2, septic shock: 2.7 (1.9–3.9); CABG: 2.7 (2.1–3.7); MAS: 2.7 (2.1–3.9); CTRL: 1.6 (1–2); CTRL vs. septic shock: *p* = 0.001; CTRL vs. CABG: *p* < 0.001; CTRL vs. MAS: *p* < 0.001). Preoperative values of both surgical groups were significantly higher compared to those of the matched control group (Figure 3, Table 2, CTRL: 1.6 (1–2); CABG: 2 (1.7–2.6); MAS: 2.6 (1.7–3.3); CTRL vs. CABG preoperative: *p* = 0.034; CTRL vs. MAS preoperative: *p* = 0.004; CABG preoperative vs. MAS preoperative: *p* = 0.354). Septic shock patients showed a significant increase at onset and over three days compared to their matched control patients (Figure 3, Table 2, septic shock onset: 3.2 (2.3–4.2); septic shock 24 h: 2.5 (1.8–3.7); septic shock 72 h: 2.3 (1–3.8); CTRL vs. septic shock onset: *p* < 0.001; CTRL vs. septic shock 24 h: *p* = 0.02; CTRL vs. septic shock 72 h: *p* = 0.05). In cardiac surgical patients, the amount of free-circulating NETs peaked immediately after the surgery and decreased significantly after 24 and 72 h, respectively (Figure 3, Table 2, CABG preoperative: 2 (1.7–2.6); CABG postoperative: 3.5 (2.7–4.6); CABG 24 h: 2.7 (2.1–3.5); CABG 72 h: 2.8 (2.1–3.8); CABG preoperative vs. CABG postoperative: *p* < 0.001; CABG postoperative vs. CABG 24 h: *p* = 0.0014; CABG postoperative vs. CABG 72 h: *p* = 0.01). MAS led to the lowest increase of NETs but gained statistical significance immediately after surgery (Figure 3, Table 2, MAS preoperative: 2.6 (1.7–3.3); MAS postoperative: 2.9 (2.3–5.2); MAS 24 h: 2.6 (2–3.8); MAS 72 h: 2.7 (2.3–3.9); MAS preoperative vs. MAS postoperative: *p* = 0.03). The postsurgical levels of free-circulating NETs did not differ compared to septic shock patients (Figure 3, Table 2).

### 3.2. Association of NETs to Inflammatory Parameters

Compared to the control group, plasma levels of MPO and IL-8 increased significantly, beginning from the onset of septic shock, and remained significantly elevated over 24 h (Table 2, MPO: CTRL vs. septic shock onset: *p* < 0.01; CTRL vs. septic shock 24 h: *p* < 0.01; CTRL vs. septic shock 72 h: *p* = 0.12; IL-8: CTRL vs. septic shock onset: *p* < 0.001; CTRL vs. septic shock 24 h: *p* < 0.01; CTRL vs. septic shock 72 h: *p* = 0.58). While MPO showed a significant postoperative increase only in MAS patients (Table 2, preoperative vs. postoperative: *p* = 0.02; preoperative vs. 24 h: *p* = 0.004), no detectable changes were found in CABG patients. With the exception of a significant elevation of IL-8 immediately after CABG, similar results were found for IL-8 expression in CABG patients (Table 2, CABG preoperative vs. postoperative: *p* = 0.008), while MAS patients presented a significant postoperative increase in IL-8 (Table 2, MAS preoperative vs. postoperative: *p* = 0.008, preoperative vs. 24 h: *p* = 0.05). Compared to the control group, changes of HMGB1 levels in septic shock patients almost reached statistical significance (at the onset of septic shock), but showed a significant increase after 24 and 72 h after septic shock onset (Table 2, control vs. septic shock onset: *p* = 0.08, control vs. septic shock 24 h *p* = 0.07; septic shock onset vs. septic shock 24 h: *p* = 0.04; septic shock onset vs. 72 h: *p* = 0.04). The other study groups did not present significant changes in HMGB1 plasma levels (Table 2).

While plasma levels of MPO and IL-8 did not correlate with the amount of free-circulating NETs in any study group, plasma levels of HMGB1 in septic shock patients showed a positive correlation to NETs (Table 3, *r* = 0.3; *p* = 0.03). Free-circulating NETs did not correlate to plasma levels of CRP and PCT nor to the leucocyte count (Table 3).

### 3.3. Association of NETs to Coagulatory Parameters

**Figure 4:**
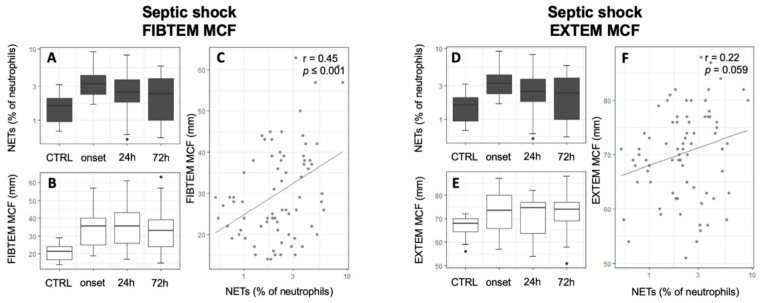
Positive correlation of free-circulating NETs to FIBTEM MCF in septic shock patients. Time courses of NETs (**A**,**D**), FIBTEM MCF (**B**), and EXTEM MCF (**E**) are shown as boxplot diagrams while scatter plots are uses to present correlations between NETs and FIBTEM MCF (**C**) and EXTEM MCF (**F**). Abbreviations: CTRL: Control group; MCF: Mean Clot Firmness; NETs: Neutrophil Extracellular Traps; *r*: Pearson’s Correlation Coefficient.

**Figure 5:**
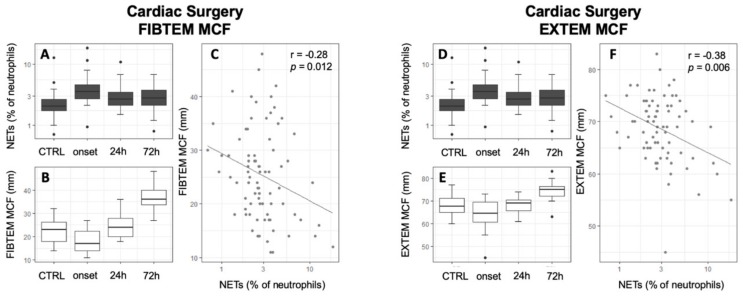
Inverse correlation of free-circulating NETs to FIBTEM MCF in cardiac surgical patients. Time courses of NETs (**A**,**D**), FIBTEM MCF (**B**), and EXTEM MCF (**E**) are shown as boxplot diagrams while scatter plots are used to present correlations between NETs and FIBTEM MCF (**C**) and EXTEM MCF (**F**). Abbreviations: CTRL: Control group; MCF: Mean Clot Firmness; NETs: Neutrophil Extracellular Traps; r: Pearson’s Correlation Coefficient.

**Figure 6:**
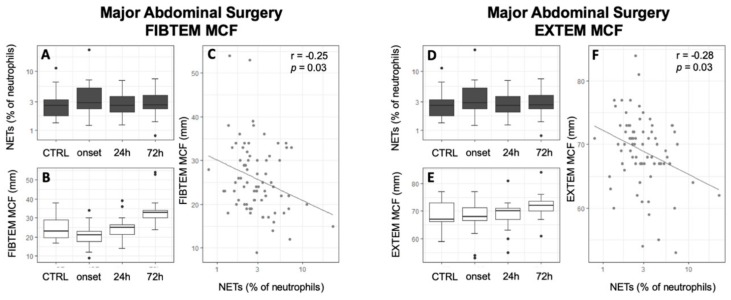
Inverse correlation of free-circulating NETs to FIBTEM MCF in major abdominal surgical patients. Time courses of NETs (**A**,**D**), FIBTEM MCF (**B**), and EXTEM MCF (**E**) are shown as boxplot diagrams while scatter plots are uses to present correlation between NETs and FIBTEM MCF (**C**) and EXTEM MCF (**F**). Abbreviations: CTRL: Control group; MCF: Mean Clot Firmness; NETs: Neutrophil Extracellular Traps; r: Pearson’s Correlation Coefficient.

In the initial analysis of the association of NETs to global coagulatory parameters, no significant correlation between NETs and PTT, INR, and fibrinogen was revealed for any study group. However, thrombocytes were positively correlated with NETs in septic shock patients (Table 3, *r* = 0.39, *p* = 0.004). While CTRL patients did not show any alterations of thrombelastographic parameters, septic shock patients showed a statistically significant negative correlation of the FIBTEM CT to free-circulating NETs and a significant positive association with the MCF (Table 3, Figure 4, FIBTEM CT: *r* = −0.3, *p* = 0.02; FIBTEM MCF: *r* = 0.37; *p* < 0.01). Other thrombelastographic assays did not reach statistical significance in septic shock patients (Supplement 4 in Supplementary Materials). Contrarily, after abdominal and cardiac surgery, a significant negative correlation of MCF with NETs could be detected in almost all assays (Table 3, Figure 5 and Figure 6). Furthermore, cardiac surgical patients showed a significant correlation of NETs with prolonged INTEM CT and CFT as well as EXTEM CFT (INTEM CT: *r* = 0.24; *p* = 0.04; INTEM CFT: *r* = 0.26; *p* = 0.02; EXTEM CFT: *r* = 0.31; *p* < 0.01). Patients who underwent MAS showed the same tendencies but only reached statistical significance in the association of EXTEM and FIBTEM CFT to NETs (EXTEM CFT: *r* = 0.27; *p* = 0.02; FIBTEM CFT: *r* = 0.25; *p* = 0.05). A significant association of NETs to a reduced LI60 could be shown in CABG and septic shock patients; however, the amount of change was minimal (Table 3, Supplement 1 in Supplementary Materials; FIBTEM LI60 septic shock: r = −0.36; *p* < 0.01; NATEM LI60 CABG: *r* = −0.32, *p* < 0.001; EXTEM LI60 CABG: *r* = −0.25, *p* = 0.03). The results of whole blood ristocetin-induced platelet impedance aggregometry did not reveal any correlations with NETs (Table 3). Furthermore, NETs did not correlate with the results of the SAC score (*r* = 0.07, *p* = 0.64).

### 3.4. Association of NETs to Outcome Parameters

Neither the SOFA scores of septic and surgical patients nor the in-hospital death rates of septic shock patients correlated with the number of free-circulating NETs (SOFA: septic shock: *r* = −0.1, *p* = 0.49; CABG: *r* = 0.17, *p* = 0.36; MAS: *r* = −0.2, *p* = 0.38; in-hospital death: septic shock: *r* = 0.05, *p* = 0.46).

## 4. Discussion

This explorative proof-of-concept study evaluated a novel flow cytometry-based approach of quantifying free-circulating NETs in an intensive care setting. Furthermore, to our knowledge, this study is the first study to directly compare NET generation in patients suffering from septic shock to that in those suffering from surgery-induced inflammation. In accordance with other studies that have used various methods to investigate NET release, flow cytometry was able to prove a significant elevation of free-circulating NETs in septic shock and postsurgical patients [18,19,26,27,31,33]. However, flow cytometry-based NET quantification did not show significant differences in the NET release between septic and postsurgical sterile systemic inflammation within the first three days following surgery and at septic shock onset. Although sepsis in the early postsurgical phase remains a challenging and severe complication, little data is available on NETosis in postsurgical infections [18,26,27,35,47]. Some studies investigated SIRS following cardiopulmonary bypass and showed an elevation in NETs after cardiac surgery, thereby supporting our study results [34,35,48]. Furthermore, trauma-induced SIRS and SIRS caused by medical conditions were associated with increased blood levels of free-circulating NETs [18,26,27]. However, to our knowledge, no other study has addressed the question of whether the degree of NETosis differs between cases of sterile postsurgical systemic inflammation and septic shock. Therefore, we directly compared the number of free-circulating NETs in septic and surgical patients and found no differences between these patient cohorts. It must be highlighted that this study was not designed to evaluate NETs as a potential biomarker; however, the results of our study question the value of flow cytometry-based NET quantification for the detection of septic complications after cardiac surgery and MAS. This may be caused by the selection of patients in our study. While all cardiac surgical patients suffered from arteriosclerosis, a high proportion of patients undergoing MAS displayed a history of malignant diseases. In contrast, a smaller proportion of septic and matched control patients suffered from arteriosclerosis and cancer. Since both diseases are associated with NETosis, baseline levels of free-circulating NETs were higher in the surgical groups compared to the (septic shock) matched controls [49,50]. Therefore, these underlying diseases may mask a relatively higher increase of NET release in septic patients. Furthermore, NETs are known to consist of nucleic acids (e.g., mitochondrial DNA), histones, platelets, and other damage-associated patterns, making them potent proinflammatory components, which are also released during cardiac surgery, trauma, and medical SIRS [26,27,34,35,48,51]. Lastly, not only might the amount of free-circulating NETs cause the association of NETs to adverse outcomes in septic surgical patients, but it may be also affected by the patient’s neutrophils’ capability to release NETs. Abram et al. induced NETosis with PMA in the blood samples of septic shock patients and showed a significantly higher capacity of releasing NETs in neutrophils deriving from septic patients compared to those from non-septic, critically ill patients [18]. Future studies must investigate whether patients suffering from postsurgical inflammation also depict a reduced capacity for PMA-induced NETosis.

Since NETs are associated with sepsis-associated coagulopathy, our study also aimed to investigate whether the amount of free-circulating NETs is associated with clinically relevant coagulopathies in septic shock and postsurgical systemic inflammation [15,18,20,21,52,53]. In accordance with Yang et al., we were able to show a procoagulatory effect of NETs in septic shock patients [19]. Yang et al. used fluorescence microscopy for NET quantification while coagulation was assessed with the measurement of thrombin–antithrombin complexes and fibrin formation. Their results showed an NET-induced hypercoagulation in septic patients. The detailed mechanisms leading to activation of coagulation during sepsis are yet not well understood; however, a tight connection between NETs, thrombin and platelet activation, and endothelium adhesion has already been described [14,15,23,54,55]. Our study focused on thrombelastographic analyses in order to reflect a high practicability for intensive care physicians and revealed a positive correlation of the number of NETs to clot firmness in fibrinogen-dependent assays as well as to the number of platelets in septic shock patients. Interestingly, in contrast to other publications, we were not able to find a correlation between NETs and the results of SAC scoring, which might be explained by our limited number of patients [18,20]. Furthermore, this study also revealed a significant negative correlation of NETs with FIBTEM and EXTEM MCF in postsurgical patients. Although interpretation of coagulatory status following cardiopulmonary bypass should be performed with caution, these results remain resilient. First, thrombelastography and global coagulatory parameters did not show severe alterations after cardiopulmonary bypass, and second, similar associations of NETs to reduced coagulatory function were also measured in MAS patients who were at low risk for postsurgical coagulopathy. Since this was the first description of this phenomena and this study did not investigate the underlying causalities, the reasons behind these associations remain unclear. Noubouossie et al. recently showed that individual histone proteins and nucleic acids, rather than NETs, directly activate the coagulatory system. The authors assumed that the procoagulatory effect of negatively charged nucleic acids might be neutralized by the histone–DNA complexes [56]. Due to the fact that bacteria can not only stimulate NETosis but also trigger the release of free-circulating nucleic acids and histones as well as directly activate the coagulatory system, the findings of Noubouossie et al. may play a role in the lack of pathogens in postsurgical SIRS [14,56,57].

Analogous to the original descriptions of this method, our validation experiments showed a positive proof of PMA-induced NET formation in flow cytometry and fluorescence microscopy [40,43]. Based on the validation experiments, we adjusted the original flow cytometry protocol of Lee et al. [43]. First, we used directly conjugated antibodies in order to further simplify the method and to reduce the risk of background staining, and second, gating was performed using the FMO technique after blocking unspecific binding sites with immunoglobin G instead of isotype controls. We chose to use the FMO technique in order to reduce the fluorescence spillover from other channels caused by the use of multiple colors, and thereby minimize errors in compensation. Furthermore, FMO offers detailed discrimination of stained cell populations while isotypes might not stain specifically. In our opinion, this adjusted protocol represents an investigator-independent and practicable approach for fast and reliable quantification of NETs which is practicable in an intensive care setting. However, flow cytometry has its limitations: First, due to the morphologic changes of neutrophils during NETosis, it should be recognized that flow cytometry may not be able to detect all NET-releasing neutrophils; in particular, swollen and degrading cells may fall out of the scatter range [41]. Therefore, later stages of NETosis might not be detected by flow cytometry. Second, with the exception of serum-based samples, blood samples must be processed immediately in order to minimize neutrophil autoactivation and cannot be frozen. Third, correlation analysis to other serum plasma surrogate parameters revealed a significant positive correlation of NETs to HMGB1 only in septic shock patients while neither HMGB1 in surgical patients nor MPO in any of the other study groups showed an association with the number of NETs as measured using flow cytometry-based quantification. This may be caused by the lack of an increase of HMGB1 in surgical patients while MPO plasma release underlies a high number of influencing factors, such as arteriosclerosis as well as systemic heparin-application, which is highly prevalent in severely ill and cardiac surgical patients [58,59]. Although NETs are induced by IL-8 via the mitogen-activated protein kinase pathway, we were not able to detect a correlation of IL-8 with circulating NETs [18]. This may be caused by a rapid decrease of IL-8 plasma levels or a varying expression of IL-8 caused by unknown influencing factors.

Furthermore, our study has other limitations: First, due to the proof-of-concept study design, we did not perform a sample size calculation. This may offer an explanation as to why this study failed to correlate the number of quantified NETs with outcome parameters. Second, the study does not allow for a conclusion for patients suffering from sepsis without signs of shock. In order to observe a high degree of NETosis, this proof-of-concept study concentrated only on septic shock patients. However, since septic patients lacking shock symptoms are also associated with adverse outcomes, the role of NETs should be further investigated in these patients [60]. Third, until today, flow cytometry-based NETs quantification remains a method requiring high expertise and technical equipment. Therefore, from a practical and financial point of view it is not yet suitable for daily routine blood analysis. However, a recent review underlines the potential for computational and automatized flow cytometry-based quantification of NETs, which is supported by the findings of this study [61]. Fourth, this study did not investigate the causal context of NETosis within the different study groups. Finally, although therapeutic heparinization occurred only in a small number of cases, a bias effect cannot be ruled out.

## 5. Conclusions

This proof-of-concept study investigated the value of flow-cytometric NET quantification in septic shock patients as well as in patients suffering from postsurgical systemic inflammation. The methodology was able to detect NETs in a reliable manner and showed a significant increase of NETs under inflammatory conditions. However, flow cytometry-based NET quantification did not distinguish between septic shock and postsurgical inflammation, casting doubt on the discriminative power of this method. Furthermore, this study showed a clinically apparent procoagulatory shift in septic patients that was associated with the free-circulating NETs. In contrast, NETs deriving from surgical patients were negatively correlated to fibrinogen-associated thrombelastographic assays. Particularly, the association of free-circulating NETs to a procoagulatory shift in septic shock may offer a therapeutic target worthy of further research. In summary, flow cytometry offers a practicable solution for the quantification of NETs in an intensive care setting. Further investigations are necessary to explain the underlying mechanisms leading to the opposing coagulatory reactions in septic and postsurgical inflammation.

## Figures and Tables

**Table 1:** Description of the study cohorts.

	Septic Shock (*n* = 20)	Cardiac Surgery (CABG, *n* = 20)	Major Abdominal Surgery (MAS, *n* = 20)	Control Patients (CTRL, *n* = 20)
General Characteristics				
Age (years)	69 (64.3–74)	70 (62–79)	68 (54–70)	69 (66.3–74.3)
Sex (% male)	70	75	60	70
BMI (kg·m^−2^)	27.9 (21.7–32.6)	30 (27.6–36.5)	24 (22.4–26.9)	27 (23.2–29.2)
ASA I II III IV V	0 0 10 (50%) 9 (45%) 1 (5%)	0 0 18 (90%) 2 (10%) 0	1 (5%) 8 (40%) 11 (55%) 0 0	1 (5%) 6 (30%) 13 (65%) 0 0
SOFA onset	10.5 (10–12.5)	NA	NA	NA
SOFA 24 h	11.5 (8–13)	3 (1–3.8)	2 (0–3)	NA
SOFA 72 h	9 (5.5–14.5)	3.5 (1–4.8)	3.5 (1.8–4.8)	NA
Focus of infection Abdominal Pulmonary Urological Soft tissue	12 (60%) 3 (15%) 3 (15%) 2 (10%)	NA	NA	NA
Type of abdominal surgery Whipple Procedure Open Partial colectomy Esophagus resection Other major abdominal surgery	NA	NA	8 (40%) 4 (20%) 4 (20%) 4 (20%)	NA
Duration of Cardiopulmonary bypass	NA	93 (74.8–111)	NA	NA
In-hospital death (%)	35	0	5	0
Preexisting Diseases				
Diabetes mellitus	9 (45%)	12 (60%)	1 (5%)	8 (40%)
Chronic kidney failure	4 (20%)	5 (25%)	1 (5%)	3 (15%)
Arteriosclerosis	14 (70%)	20 (100%)	5 (25%)	14 (70%)
Malignant cancerous disease	7 (35%)	0	13 (65%)	7 (35%)
Anticoagulatory Therapy				
Prophylactic heparinization onset/preoperative	10 (50%)	20 (100%)	20 (100%)	15 (75%)
Prophylactic heparinization postoperative		0	0	
Prophylactic heparinization 24 h	12 (60%)	18 (90%)	18 (90%)	
Prophylactic heparinization 72 h	11 (55%)	16 (80%)	18 (90%)	
Therapeutic heparinization onset/preoperative	8 (40%)	0	0	5
Therapeutic heparinization postoperative		0	1 (5%)	
Therapeutic heparinization 24 h	6 (30%)	2 (10%)	1 (5%)	
Therapeutic heparinization 72 h	7 (35%)	3 (15%)	1 (5%)	

Data are shown as median (interquartile range) or as an absolute number and percentage (*n* (%)) of the study group. Abbreviations: ASA: American Society of Anesthesiology Score; BMI: Body Mass Index; SOFA: Sepsis-related Organ Failure Assessment; NA: not applicable.

**Table 2:** Results of inflammatory parameters.

	Septic Shock (*n* = 20)		Cardiac Surgery (CABG, *n* = 20)		Major Abdominal Surgery (MAS, *n* = 20)		Control Patients (CTRL, *n* = 20)	
**Leucocytes (L^−1^)**	onset 24 h 72 h	11.9 (7.1–19.7) 13.5 (9.3–20.9) 14.2 (10.7–17.3)	Preop Postop 24 h 72 h	8.1 (6.6–9.4) 11 (7.9–15) 10.7 (8.2–12.2) 10.6 (8.2–11.8)	Preop Postop 24 h 72 h	7.6 (6–9] 10.3 (9.4–12.5) 11.5 (9.3–12.9) 7.4 (6.5–11.6)	Ctrl	5.9 (5.3–7.9)
**CRP (mg/L)**	onset 24 h 72 h	229.5 (117.2–277.3) 244.6 (166.5–287.7) 236.5 (139.5–268.8)	Preop Postop 24 h 72 h	3.8 (1.9–10.6) 4.3 (2.6–9.2) 75.1 (67.2–109.8) 202.4 (156.3–241.2)	Preop Postop 24 h 72 h	5.1 (1.7–10.3) 6.5 (2.5–11.4) 68 (46.6–88.5) 149 (115.7–200)	Ctrl	1.1 (0–6.4)
**PCT (µg/L)**	onset 24 h 72 h	9.2 (5.2–38.1) 10.4 (4.9–29.2) 7 (2.2–25.6)	Preop Postop 24 h 72 h	0.2 (0.1–0.2) N.A. N.A 1.6 (1.6)	Preop Postop 24 h 72 h	N.A. 0.6 (0.4–0.7) 0.7 (0.3–0.9) 0.8 (0.4–0.9)	Ctrl	N.A.
**NETs (%)**	onset 24 h 72 h	3.2 (2.3–4.2) 2.5 (1.8–3.7) 2.3 (1–3.8)	Preop Postop 24 h 72 h	2 (1.7–2.6) 3.5 (2.7–4.6) 2.7 (2.1–3.5) 2.8 (2.1–3.8)	Preop Postop 24 h 72 h	2.6 (1.7–3.3) 2.9 (2.3–5.2) 2.6 (2–3.8) 2.7 (2.3–3.9)	Ctrl	1.6 (1–2)
**HMGB1 (pg/mL)**	onset 24 h 72 h	40,332.1 (25,079.6–51,674.9) 32,692.3 (21,563.6–50,421.8) 25,496.2 (23,125.4–33,421.3)	Preop Postop 24 h 72 h	25,241.3 (20,953.1–46,031.4) 23,982.5 (17,353.2–49,133.1) 30,440.2 (22,238.5–41,098.5) 26,584.3 (20,870.2–38,988.1)	Preop Postop 24 h 72 h	31,126.8 (20,032.8–38,097.8) 25,343.5 (21,913.1–41,784.2) 28,800.1 (21,687.7–39,665.6) 21,780.6 (16,867–34,755.6)	Ctrl	26,297.5 (22,149.3–34,710.9)
**MPO (ng/mL)**	onset 24 h 72 h	700,905.7 (285,135.5–886,644) 542,611.2 (303,891–832,728.9) 498,553 (381,058.9–610,573.3)	Preop Postop 24 h 72 h	392,102.8 (199,581–571,528,04) 438,502.8 (341,657.5–638,995.4) 595,820.4 (275,593.4–892,010.7) 529,317.3 (306,869.6–885,046)	Preop Postop 24 h 72 h	367,381.5 (187,582–499,310.8) 480,111 (344,182.5–885,513.8) 713,023.1 (433,356.9–913,219.4) 351,888,.5 (235,179.9–711,455.7)	Ctrl	214,472.6 (136,124.2–296,626.7)
**Interleukin 8 (pg/mL)**	onset 24 h 72 h	470.4 (105.9–1462,30) 206.6 (100.1–489.9) 165.1 (90.2–195.5)	Preop Postop 24 h 72 h	39.2 (26.1–49) 85.3 (57.7–127.9) 67.1 (40.7–99) 55.2 (42.9–72.2)	Preop Postop 24 h 72 h	35 (20.4–49.8) 71.1 (58.2–129.1) 60.9 (41.2–110.4) 41.9 (27.1–63.6)	Ctrl	35.8 (25–40.5)

Data are shown as median (IQR). Abbreviations: CRP: C-Reactive Protein; DNA: Deoxynucleic Acid; HMGB1: High-Mobility-Group-Protein B1; MPO: Myeloperoxidase; NETs: Neutrophil Extracellular Traps; PCT: Procalcitonin.

**Table 3:** Correlation of NETs to coagulatory and inflammatory parameters.

Parameter	Septic Shock (*n* = 20)		Cardiac Surgery (CABG, *n* = 20)		Major Abdominal Surgery (MAS, *n* = 20)		Control Patients (CTRL, *n* = 20)	
	Correlation Coefficient	*p*-Value	Correlation Coefficient	*p*-Value	Correlation Coefficient	*p*-Value	Correlation Coefficient	*p*-Value
Thrombelastography								
EXTEM CFT (s)	−0.10	0.47	0.31	<0.01	0.27	0.02	0.04	0.87
FIBTEM CFT (s)	−0.15	0.31	0.00	1.00	0.25	0.05	−0.22	0.50
INTEM CFT (s)	0.07	0.61	0.26	0.02	0.20	0.09	−0.23	0.34
NATEM CFT (s)	−0.12	0.41	−0.09	0.44	−0.01	0.91	0.14	0.55
EXTEM CT (s)	−0.20	0.14	0.01	0.91	0.12	0.30	−0.43	0.06
FIBTEM CT (s)	−0.31	0.02	−0.02	0.85	0.00	0.99	−0.42	0.07
INTEM CT (s)	0.00	0.98	0.24	0.04	0.12	0.33	−0.33	0.16
NATEM CT (s)	−0.04	0.80	−0.10	0.38	−0.04	0.74	−0.06	0.80
EXTEM LI60 (%)	−0.08	0.55	−0.25	0.03	0.01	0.97	−0.02	0.94
FIBTEM LI60 (%)	−0.36	<0.01	−0.04	0.70	0.06	0.59	0.32	0.17
INTEM LI60 (%)	−0.12	0.38	−0.21	0.06	0.02	0.85	0.12	0.62
NATEM LI60 (%)	−0.16	0.30	−0.32	<0.001	0.03	0.84	0.11	0.65
EXTEM MCF (mm)	0.15	0.27	−0.38	<0.001	−0.28	<0.01	−0.25	0.28
FIBTEM MCF (mm)	0.37	≤0.01	−0.28	<0.01	−0.25	0.03	−0.38	0.10
INTEM MCF (mm)	0.18	0.19	−0.41	<0.001	−0.32	<0.01	−0.21	0.38
NATEM MCF (mm)	0.20	0.16	−0.23	0.04	-0.09	0.46	−0.33	0.15
Impedance Aggregometry								
ASPItest (Units)	0.24	0.08	0.019	0.87	−0.063	0.6	−0.1	0.67
TRAPtest (Units)	0.17	0.22	−0.058	0.61	−0.085	0.48	−0.11	0.64
ADPtest (Units)	0.07	0.64	−0.12	0.3	−0.07	0.56	−0.05	0.82
Global Coagulatory Parameters								
PTT (s)	−0.15	0.28	0.03	0.79	−0.09	0.5	0.09	0.7
INR	−0.21	0.12	0.18	0.1	0.08	0.52	0.16	0.53
Platelet count (L^−1^)	0.39	0.004	−0.032	0.78	−0.16	0.17	0.048	0.84
Fibrinogen (g/L)	0.31	0.101	−0.26	0.07	−0.1	0.7	NA	NA
Inflammatory Parameters								
Leucocytes (L^−1^)	0.007	0.96	−0.016	0.89	−0.12	0.33	−0.21	0.37
CRP (mg/L)	−0.1	0.47	−0.14	0.24	−0.12	0.34	−0.51	0.32
PCT (µg/L)	0.059	0.69	0.6	0.59	0.12	0.68	N.A.	N.A.
HMGB-1 (pg/mL)	0.30	0.03	0.04	0.76	−0.08	0.51	−0.43	0.06
MPO (ng/mL)	−0.16	0.24	0.04	0.75	−0.06	0.6	−0.19	0.41
Interleukin 8 (pg/mL)	0.01	0.93	0.16	0.16	0.04	0.70	−0.21	0.37

Data were derived from Pearson’s correlation analysis. Significant p-values are highlighted in bold. Abbreviations: CABG: Coronary Artery Bypass Graft; CRP: C-Reactive Protein; CFT: Clot Firmness Time; CT: Clotting Time; CTRL: Control group; DNA: Deoxynucleic Acid; HMGB1: High-Mobility-Group-Protein B1; INR: International normalized ratio; LI60: Lysis Index after 60 min; MAS: Major Abdominal Surgery; MCF: Mean Clot Firmness; MPO: Myeloperoxidase; NA: not applicable; NETs: Neutrophil Extracellular Traps; PCT: Procalcitonin; PTT: Partial Thromboplastin Time.

## Supplementary Materials

The following are available online at https://www.mdpi.com/2077-0383/9/1/174/s1, Supplement 1 Demonstration of preliminary tests with 7-AAD in order to prove vitality of neutrophils (red box). First, leucocytes were targeted (**A**) and identified as CD15^+^-cells (**B**). Then, MPO- and anti-H3-Histone-positive cells were defined as surrogates for NETs (**C**). 7-AAD negative cells represent vital cells (**D**) and are quantified to a high proportion (**E**). Abbreviations: APC: Allophycocyanin; FITC: Fluorescein isothiocyanate; FSC: Forward Scatter; MPO: Myeloperoxidase; NETs: Neutrophil Extracellular Traps; SSC: Side Scatter, 7-AAD: 7-aminoactinomycin. Supplement 2 Comparison of FMO and isotype controls. FMO (**A**,**C**) and isotypes (**B**,**D**) show comparable gating results. PMA leads to a strong activation of NET release (**E**). Results are shown in percentage of gated neutrophils (**F**). Abbreviations: APC: Allophycocyanin; FITC: Fluorescein isothiocyanate; FMO: Fluorescence-minus-one; FSC: Forward Scatter; MPO: Myeloperoxidase; NETs: Neutrophil Extracellular Traps; PMA: phorbol 12-myristate 13-acetate; SSC: Side Scatter. Supplement 3 Fluorescence microscopy of native and PMA stimulated neutrophils. Native neutrophils are shown in the upper row with marked stainings (**A**–**C**), while in the lower row, NET-releasing neutrophils are shown after PMA stimulation with the accordant stainings (**D**–**F**). NETs show a typical comet tail configuration. G displays the overlay of all staining antibodies. Abbreviations: MPO: Myeloperoxidase; NETs: Neutrophil Extracellular Traps; PMA: phorbol 12-myristate 13-acetate. Supplement 4 Results of thrombelastography. Data are shown as median (IQR). Abbreviations: CFT: Clot Firmness Time; CT: Clotting Time; LI60: Lysis Index after 60 min; MCF: Mean Clot Firmness.
