# Supplementary material for: Flow Cytometry-Based Quantification of Neutrophil Extracellular Traps Shows an Association with Hypercoagulation in Septic Shock and Hypocoagulation in Postsurgical Systemic Inflammation—A Proof-of-Concept Study
Source: J Clin Med. 2020 Jan 8;9(1):174. doi: 10.3390/jcm9010174 (PMC7019434; doi:10.3390/jcm9010174)
Supplement: Supplementary file 1 [file jcm-09-00174-s001.pdf]

Supplement 1 Demonstration of preliminary tests with 7-AAD in order to prove vitality of neutrophils (red box). First, leucocytes were targeted (**A**) and identified as CD15$^+$-cells (**B**). Then, MPO- and anti-H3-Histone-positive cells were defined as surrogates for NETs (**C**). 7-AAD negative cells represent vital cells (**D**) and are quantified to a high proportion (**E**). Abbreviations: APC: Allophycocyanin; FITC: Fluorescein isothiocyanate; FSC: Forward Scatter; MPO: Myeloperoxidase; NETs: Neutrophil Extracellular Traps; SSC: Side Scatter, 7-AAD: 7-aminoactinomycin.

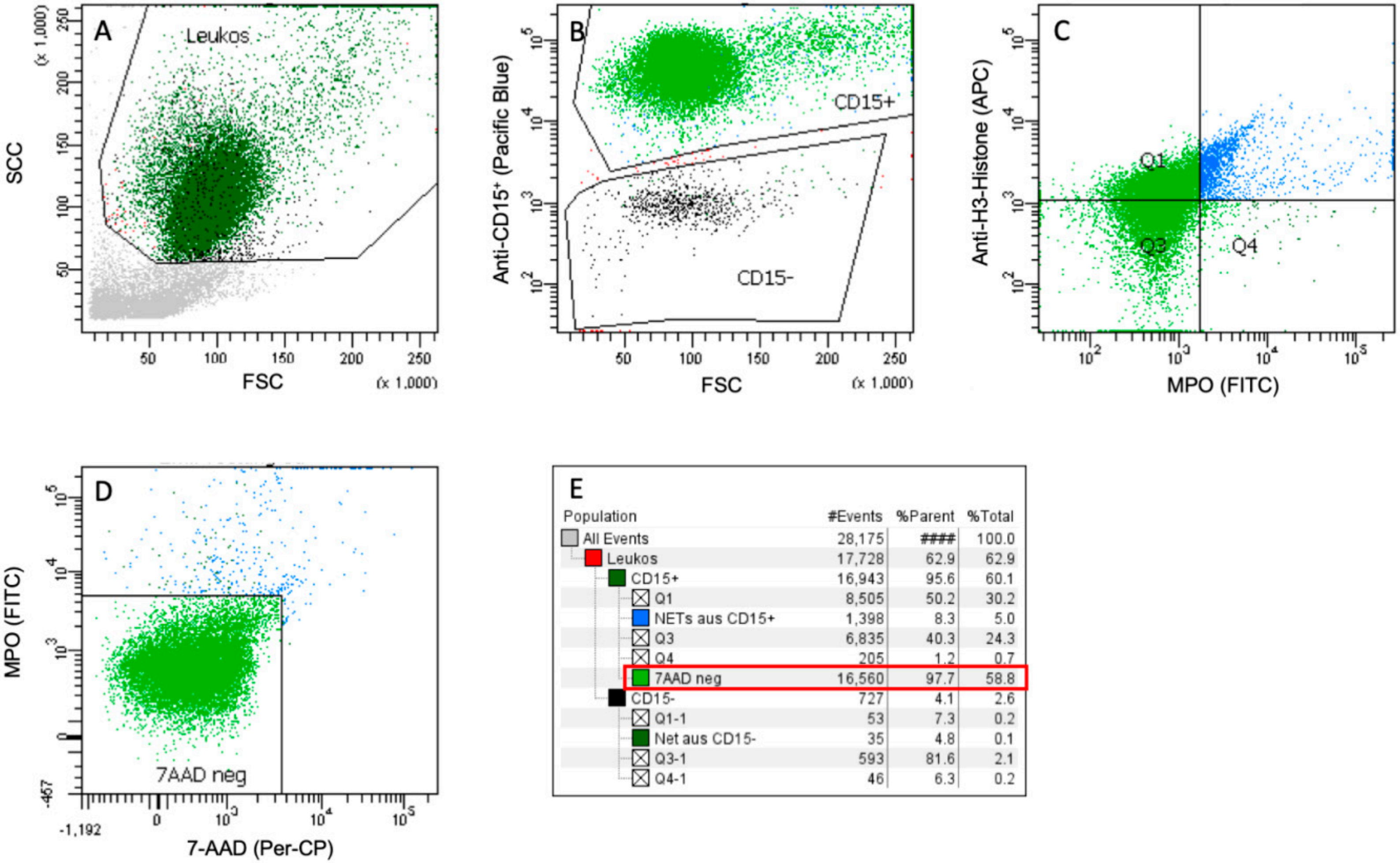

Population	#Events	%Parent	%Total
All Events	28,175	####	100.0
Leukos	17,728	62.9	62.9
CD15+	16,943	95.6	60.1
Q1	8,505	50.2	30.2
NETs aus CD15+	1,398	8.3	5.0
Q3	6,835	40.3	24.3
Q4	205	1.2	0.7
7AAD neg	16,560	97.7	58.8
CD15-	727	4.1	2.6
Q1-1	53	7.3	0.2
Net aus CD15-	35	4.8	0.1
Q3-1	593	81.6	2.1
Q4-1	46	6.3	0.2

Supplement 2 Comparison of FMO and isotype controls. FMO (**A**,**C**) and isotypes (**B**,**D**) show comparable gating results. PMA leads to a strong activation of NET release (**E**). Results are shown in percentage of gated neutrophils (**F**). Abbreviations: APC: Allophycocyanin; FITC: Fluorescein isothiocyanate; FMO: Fluorescence-minus-one; FSC: Forward Scatter; MPO: Myeloperoxidase; NETs: Neutrophil Extracellular Traps; PMA: phorbol 12-myristate 13-acetate; SSC: Side Scatter.

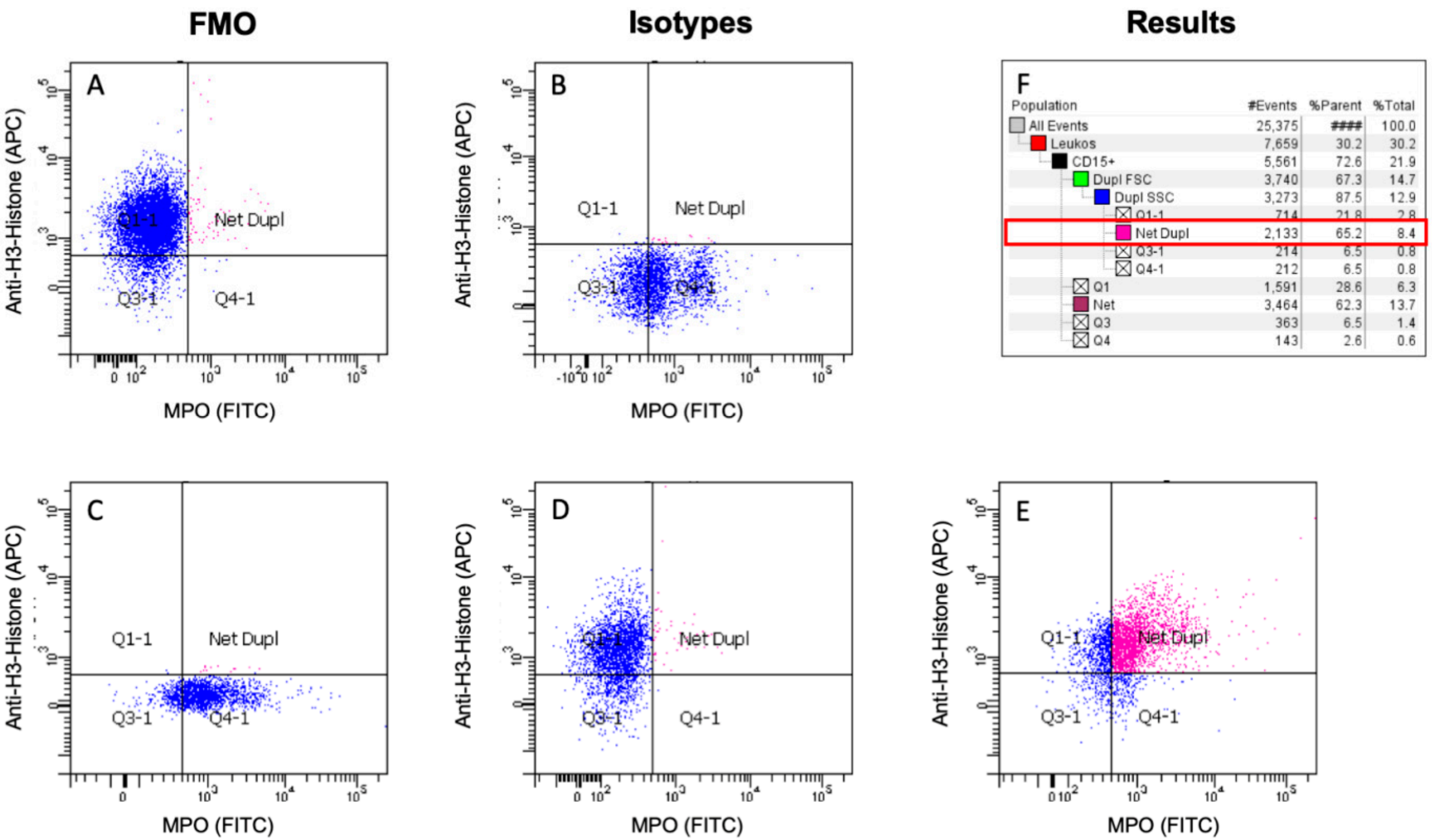

Supplement 3 Fluorescence microscopy of native and PMA stimulated neutrophils. Native neutrophils are shown in the upper row with marked stainings (**A**–**C**), while in the lower row, NET-releasing neutrophils are shown after PMA stimulation with the accordant stainings (**D**–**F**). NETs show a typical comet tail configuration. G displays the overlay of all staining antibodies. Abbreviations: MPO: Myeloperoxidase; NETs: Neutrophil Extracellular Traps; PMA: phorbol 12-myristate 13-acetate.

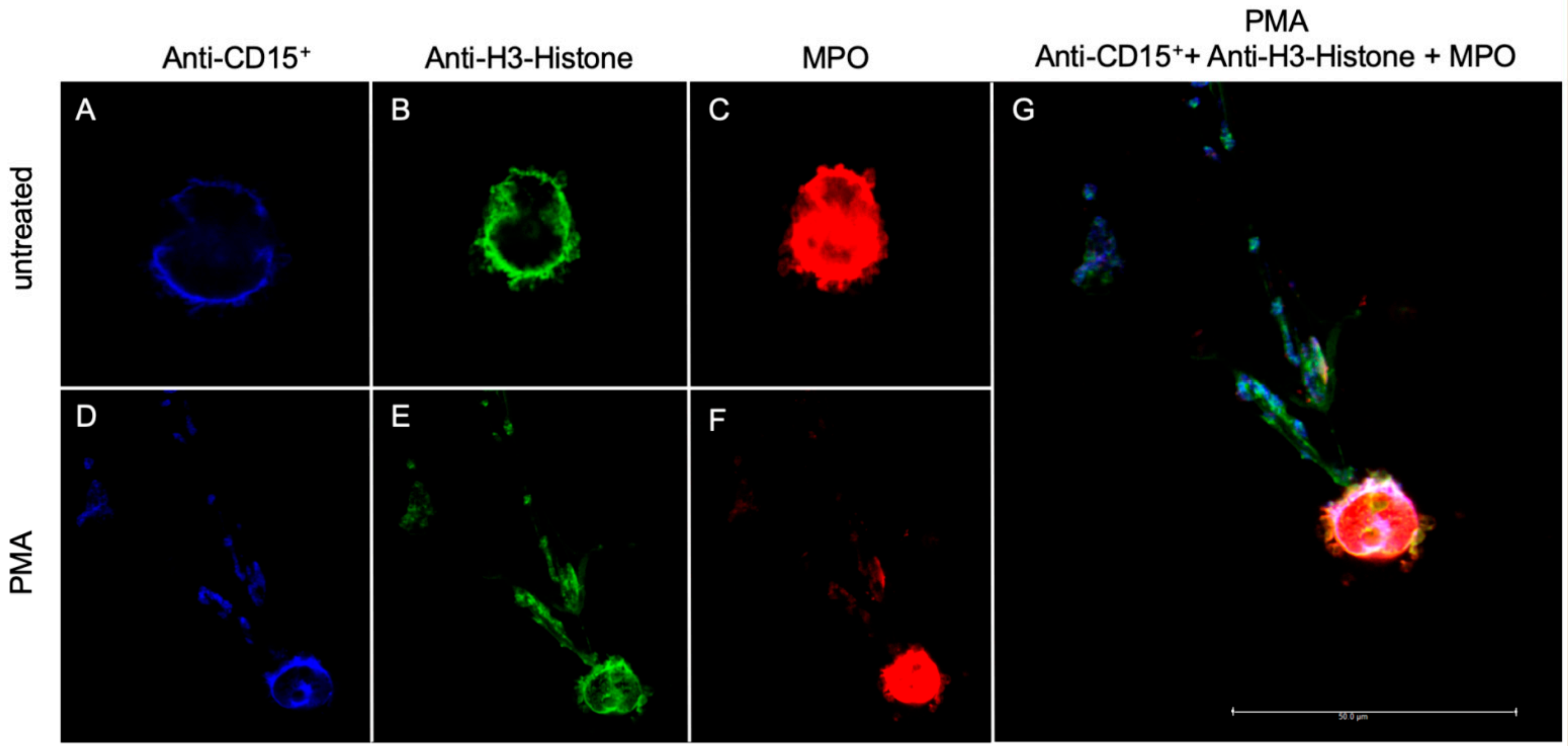

Supplement 4 Results of thrombelastography. Data are shown as median (IQR). Abbreviations: CFT: Clot Firmness Time; CT: Clotting Time; LI60: Lysis Index after 60 min; MCF: Mean Clot Firmness.

	**Septic Shock**		**Cardiac Surgery**		**Major Abdominal Surgery**		**Control Patients**	
**EXTEM CFT (sec)**	onset	44 (37.8-64.8)	Preop	55.5 (48.8-67.5)	Preop	54 (45.5-63.3)	Ctrl	60 (55.8-71.3)
	24 h	40 (36.8-68.3)	Postop	69.5 (56.5-82.5)	Postop	58 (47.8-72.3)		
	72 h	48 (40-55)	24 h	56.5 (48.5-62)	24 h	52 (48-62.5)		
			72 h	43.5 (41-46)	72 h	48 (40.5-55)		
**EXTEM CT (sec)**	onset	84 (76.3-97.5)	Preop	62.5 (58.8-67.5)	Preop	66 (60-69.3	Ctrl	68 (65-78.3)
	24 h	78.5 (67.5-96.3)	Postop	80.5 (72.8-82)	Postop	70.5 (60.8-73)		
	72 h	81 (69-100)	24 h	71.5 (67.3-79.8)	24 h	68 (57-77)		
			72 h	73.5 (61-87)	72 h	77 (62.5-85.5)		
**EXTEM LI60 (%)**	onset	95 (91.8-97)	Preop	93 (90.5-94.5)	Preop	92.5 (91-94.3)	Ctrl	92.5 (89.5-94-3)
	24 h	95 (93.5-99)	Postop	94.5 (93.8-97)	Postop	94.5 (93-96)		
	72 h	95 (94-97)	24 h	91 (90-95)	24 h	93 (90-93.8)		
			72 h	90 (90-92.3)	72 h	91 (88.5-93)		
**EXTEM MCF (mm)**	onset	73.5 (65.8-80)	Preop	67.5 (65-71.3)	Preop	67 (66.3-73)	Ctrl	68 (64.5-70)
	24 h	74.5 (63.8-77)	Postop	64.5 (60.8-69.5)	Postop	68 (66.5-71.3)		
	72 h	74 (69-77)	24 h	69 (65.8-70.5)	24 h	70 (67-71)		
			72 h	75 (72-76.3)	72 h	72 (70-73.5)		
**INTEM CFT (sec)**	onset	52.5 (39.5-82.3)	Preop	58.5 (44-66.5)	Preop	55 (46-65)	Ctrl	66.5 (53-76.3)
	24 h	48 (37-71.3)	Postop	89 (72-114.5)	Postop	53 (50-64.5)		
	72 h	51 (46-62)	24 h	59 (50-65.3)	24 h	52 (46-66)		
			72 h	45 (41.8-47)	72 h	47 (42.5-55)		
**INTEM CT (sec)**	onset	197 (164.5-262.8)	Preop	181.5 (156-193.3)	Preop	186 (134-201.3)	Ctrl	189 (150.8-215.8)
	24 h	206.5 (174-238.3)	Postop	265 (211-323)	Postop	158 (142.8-169.8)		
	72 h	219 (192-293)	24 h	193 (161.3-205.8)	24 h	171 (153-195)		
			72 h	179 (151.3-206.5)	72 h	172 (153.5-215)		
**INTEM LI60 (%)**	onset	96 (92.5-98)	Preop	92 (90-93.5)	Preop	90.5 (89-92.3)	Ctrl	90.5 (88.5-93)

	24 h<br>72 h	96 (93.5-98)<br>96 (93-99)	Postop<br>24 h<br>72 h	93 (91.5-96)<br>90 (88-93)<br>90 (88-92)	Postop<br>24 h<br>72 h	93 (91-95)<br>91 (89.3-93)<br>90 (86-91)		
**INTEM MCF (mm)**	onset<br>24 h<br>72 h	71.5 (63.8-76)<br>71.5 (63.5-77.3)<br>72 (67-75)	Preop<br>Postop<br>24 h<br>72 h	64.5 (62-68.5)<br>61 (56.5-62.5)<br>66.5 (63-68.3)<br>72 (68.8-74)	Preop<br>Postop<br>24 h<br>72 h	65.5 (62-69.3)<br>66 (65-68)<br>66 (63-69)<br>68 (66.5-70.5)	Ctrl	66 (60.8-67)
**FIBTEM CFT (sec)**	onset<br>24 h<br>72 h	64 (46.5-164)<br>59.5 (41.5-103)<br>68 (51-120)	Preop<br>Postop<br>24 h<br>72 h	137 (76-341.3)<br>285.5 (131.3-580.8)<br>121.5 (84.8-452)<br>51 (44-60.8)	Preop<br>Postop<br>24 h<br>72 h	130 (71-272.5)<br>340.5 (197.5-627.5)<br>178 (108.5-308.3)<br>65 (55.5-76.5)	Ctrl	202 (111.5-362.8)
**FIBTEM CT (sec)**	onset<br>24 h<br>72 h	67 (62.3-71)<br>77.5 (69-96)<br>76 (66-92)<br>83 (73-93)	Preop<br>Postop<br>24 h<br>72 h	60.5 (55.3-65)<br>74.5 (69.3-85.8)<br>70 (61.8-81.8)<br>68.5 (65.5-73.5)	Preop<br>Postop<br>24 h<br>72 h	62 (57.5-69.3)<br>65.5 (55.5-71)<br>68 (61-78.5)<br>68 (58.5-79)	Ctrl	214472.6 (136124.2-296626.7)
**FIBTEM LI60 (%)**	onset<br>24 h<br>72 h	100 (99-100)<br>100 (100)<br>100 (99-100)	Preop<br>Postop<br>24 h<br>72 h	100<br>100<br>100<br>100	Preop<br>Postop<br>24 h<br>72 h	100<br>100<br>100<br>100	Ctrl	100 (100)
**FIBTEM MCF (mm)**	onset<br>24 h<br>72 h	35.5 (25-40)<br>35.5 (26-43.3)<br>33 (24-39)	Preop<br>Postop<br>24 h<br>72 h	23 (18-26.3)<br>17 (14-22.3)<br>24 (20-28)<br>36 (33.8-40)	Preop<br>Postop<br>24 h<br>72 h	23 (19.8-29)<br>21 (17.8-23)<br>25 (21.5-26.5)<br>33 (30-34)	Ctrl	21.5 (16.8-24)
**NATEM CFT (sec)**	onset<br>24 h<br>72 h	209 (164.5-388.5)<br>188 (120-344)<br>256 (155-543)	Preop<br>Postop<br>24 h<br>72 h	144.5 (123-214.3)<br>202 (150-244)<br>220 (143.5-291.8)<br>172.5 (132.5-383.3)	Preop<br>Postop<br>24 h<br>72 h	139.5 (106-150.3)<br>118.5 (87.8-201.5)<br>155 (131.5-236)<br>129.5 (94.3-220.3)	Ctrl	153.5 (143.3-185.8)
**NATEM CT (sec)**	onset	719 (529.5-1009)	Preop	629 (516.3-700.3)	Preop	529.5 (507.8-627.5)	Ctrl	639.5 (544.5-678.3)

	24 h	757 (561-889)	Postop	643 (604.5-801.5)	Postop	474 (382-597.5)		
	72 h	830 (546-1030)	24 h	619.5 (550.3-769)	24 h	611 (536-687)		
			72 h	603.5 (559.8-767)	72 h	497 (455-774.5)		
**NATEM LI60 (%)**	onset	99 (95-100)	Preop	92.5 (89.2-95)	Preop	91.5 (90-94.3)	Ctrl	92 (92-95)
	24 h	99 (96.5-100)	Postop	94 (91-95)	Postop	95 (93-96.3)		
	72 h	99 (96.5-100)	24 h	92 (90-96)	24 h	92 (90-93.3)		
			72 h	94.5 (91-98.3)	72 h	92 (89-96)		
**NATEM MCF (mm)**	onset	67 (56.5-73)	Preop	58 (55.8-63.3)	Preop	61.5 (56.8-66.5)	Ctrl	58.5 (56.8-62.3)
	24 h	67 (53.5-78)	Postop	56 (47-59.5)	Postop	61 (56.3-66)		
	72 h	71 (55-75)	24 h	60.5 (56-64.5)	24 h	61 (55.5-64.5)		
			72 h	70 (67-77)	72 h	69 (64.5-72.5)		
